# [μ-10,21-Dimethyl-3,6,14,17-tetra­za­tricyclo­[17.3.1.1^8,12^]tetra­cosa-1(23),2,6,8,10,12 (24),13,17,19,21-deca­ene-23,24-diolato-κ^4^ *N* ^3^,*N* ^6^,*O* ^23^,*O* ^24^:κ^4^ *N* ^14^,*N* ^17^,*O* ^23^,*O* ^24^]bis­(perchlorato-κ*O*)dimanganese(II)

**DOI:** 10.1107/S1600536808035551

**Published:** 2008-11-08

**Authors:** Jing Liu, Zhi-Quan Pan, Hong Zhou, Yi-Zhi Li

**Affiliations:** a Key Laboratory for Green Chemical Processes of the Ministry of Education, Wuhan Institute of Technology, Wuhan 430073, People’s Republic of China; b State Key Laboratory of Coordination Chemistry, Coordination Chemistry Institute, Nanjing University, Nanjing 210093, People’s Republic of China

## Abstract

In the centrosymmetric and dinuclear title complex, [Mn_2_(C_22_H_22_N_4_O_2_)(ClO_4_)_2_], the two Mn atoms are bridged by two phenolate O atoms of the N_4_O_2_ macrocycle with an Mn⋯Mn distance of 2.9228 (11) Å. The distorted square–pyramidal N_2_O_3_ coordination geometry is completed by an O atom derived from a perchlorate anion.

## Related literature

For related literature, see: Bai *et al.* (2007 ▶); Venegas-Yazigi *et al.* (2006 ▶); Jong *et al.* (2006 ▶); Ki *et al.* (2006 ▶); Tei *et al.* (2001 ▶); Brooker & Croucher (1997 ▶); Chattopadhyay *et al.* (2007 ▶). For synthesis, see: Taniguchi (1984 ▶). 
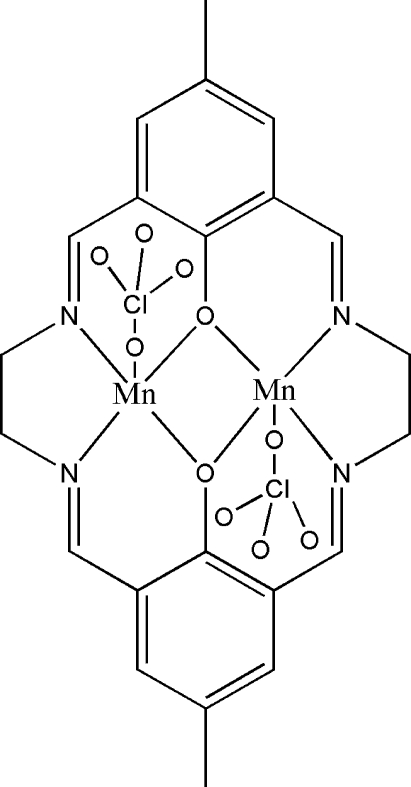


## Experimental

### 

#### Crystal data

[Mn_2_(C_22_H_22_N_4_O_2_)(ClO_4_)_2_]
*M* *_r_* = 683.22
Triclinic, 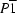

*a* = 8.3129 (10) Å
*b* = 8.3759 (11) Å
*c* = 9.9712 (12) Å
α = 81.484 (2)°
β = 68.520 (3)°
γ = 78.838 (2)°
*V* = 631.56 (14) Å^3^
*Z* = 1
Mo *K*α radiation
μ = 1.28 mm^−1^
*T* = 291 (2) K
0.31 × 0.21 × 0.15 mm

#### Data collection

Bruker SMART APEX CCD diffractometer
Absorption correction: multi-scan (*SADABS*; Bruker, 2000 ▶) *T*_min_ = 0.73, *T*_max_ = 0.83
3663 measured reflections
2439 independent reflections
1701 reflections with *I* > 2σ(*I*)
*R*_int_ = 0.029

#### Refinement

*R*[*F* ^2^ > 2σ(*F* ^2^)] = 0.052
*wR*(*F* ^2^) = 0.118
*S* = 0.99
2439 reflections
182 parameters
H-atom parameters constrained
Δρ_max_ = 0.56 e Å^−3^
Δρ_min_ = −0.58 e Å^−3^

### 

Data collection: *SMART* (Bruker, 2000 ▶); cell refinement: *SAINT* (Bruker, 2000 ▶); data reduction: *SAINT*; program(s) used to solve structure: *SHELXTL* (Sheldrick, 2008 ▶); program(s) used to refine structure: *SHELXTL*; molecular graphics: *SHELXTL*; software used to prepare material for publication: *SHELXTL*.

## Supplementary Material

Crystal structure: contains datablocks global, I. DOI: 10.1107/S1600536808035551/tk2317sup1.cif

Structure factors: contains datablocks I. DOI: 10.1107/S1600536808035551/tk2317Isup2.hkl

Additional supplementary materials:  crystallographic information; 3D view; checkCIF report

## Figures and Tables

**Fig. 1.**
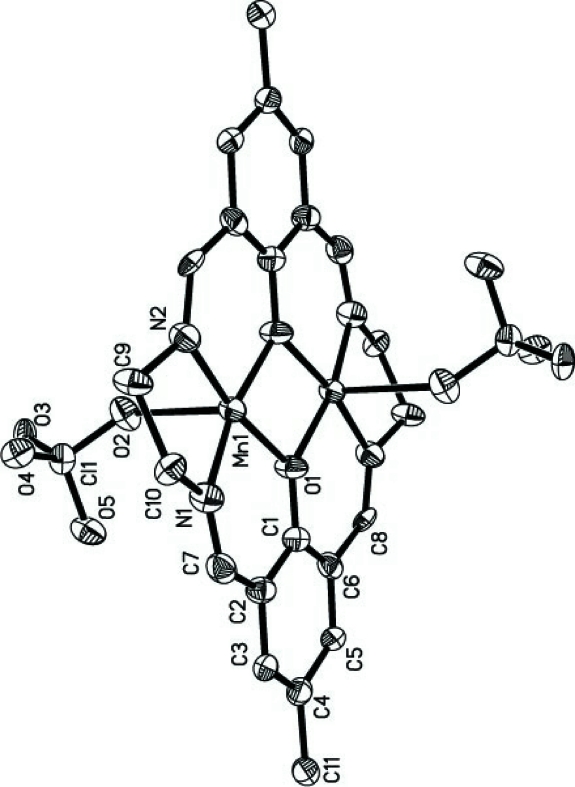
A view of (I), showing the labeling of the non-H atoms and 30% probability ellipsoids. H atoms have been omitted for clarity.

## supplementary crystallographic information

### Comment

Schiff base macrocyclic complexes, derived from the cyclocondensation of 2,6-di-formyl-4-phenol and alkylenediamine in the presence of metal ions, have been extensively studied (Ki *et al.*, 2006; Brooker & Croucher, 1997). The properties of the complexes vary with the differences in the macrocyclic structures and in the nature of the metal ions (Tei *et al.*, 2001; Jong *et al.*, 2006; Venegas-Yazigi *et al.*, 2006). Although the same macrocyclic ligand featured in the title complex, (I), exists in the literature (Bai *et al.*, 2007; Chattopadhyay *et al.*, 2007), the dinuclear Mn(II) complex is novel; the structure is reported herein.

The dinuclear and centrosymmetric structure of (I), Fig. 1, is constructed about a Mn_2_O_2_ core. The macrocyclic ligand is hexadentate forming an N_4_O_2_ donor set. The Mn ion is coordinated by two endogenous phenolic-O atoms and two azomethine-N atoms that form an approximately square planar geometry. The distorted square pyramidal geometry is completed by a weakly coordinated O atom derived from the perchlorate anion, 2.390 (3) Å. The latter distance is greater than the range of the other Mn-(donor atom) distances, i.e. 1.888 (3) to 1.909 (3) Å. The Mn—Mn distance is 2.9228 (11) Å.

### Experimental

2,6-Di-formyl-4-methylphenol was prepared according to the literature method (Taniguchi, 1984). Ethylenediamine (0.8 mmol, 0.048 g) in absolute methanol (10 ml) was added to a methanol solution (10 ml) containing 2,6-di-formyl-4-methylphenol (0.8 mmol, 0.13 g). The solution was stirred vigorously for 3 h in a ice-bath. Afterwards, a methanol solution (5 ml) of Mn(OAc)_2_.4H_2_O (0.4 mmol, 0.1 g) was added dropwise over a period of 1 h at room temperature. The mixture was stirred for a further 12 h at ambient temperature. Finally, Mn(ClO_4_)_2_.6H_2_O (0.4 mmol, 0.15 g) dissolved in methanol (5 ml) was added to the mixture and stirred for 8 h at room temperature. The dark-red block-shaped crystals suitable for X-ray diffraction precipitated by slow volatilization over a period of one month.

### Refinement

All C-bound H atoms were placed in calculated positions with 0.93–0.97 Å, and included in the refinement in the riding-model approximation, with *U*(H) set to 1.2–1.5*U*_eq_(C).

### Crystal data

**Table:**

[Mn_2_(C_22_H_22_N_4_O_2_)(ClO_4_)_2_]	*Z* = 1
*M**_r_* = 683.22	*F*_000_ = 346
Triclinic, *P*1	*D*_x_ = 1.796 Mg m^−^^3^
Hall symbol: -P 1	Mo *K*α radiation λ = 0.71073 Å
*a* = 8.3129 (10) Å	Cell parameters from 1608 reflections
*b* = 8.3759 (11) Å	θ = 2.5–25.7º
*c* = 9.9712 (12) Å	µ = 1.28 mm^−^^1^
α = 81.484 (2)º	*T* = 291 (2) K
β = 68.520 (3)º	Block, red
γ = 78.838 (2)º	0.31 × 0.21 × 0.15 mm
*V* = 631.56 (14) Å^3^	

### Data collection

**Table:**

Bruker SMART APEX CCD diffractometer	2439 independent reflections
Radiation source: sealed tube	1701 reflections with *I* > 2σ(*I*)
Monochromator: graphite	*R*_int_ = 0.029
*T* = 291(2) K	θ_max_ = 26.0º
φ and ω scans	θ_min_ = 2.2º
Absorption correction: multi-scan(SADABS; Bruker, 2000)	*h* = −6→10
*T*_min_ = 0.73, *T*_max_ = 0.83	*k* = −9→10
3663 measured reflections	*l* = −12→12

### Refinement

**Table:**

Refinement on *F*^2^	Secondary atom site location: difference Fourier map
Least-squares matrix: full	Hydrogen site location: inferred from neighbouring sites
*R*[*F*^2^ > 2σ(*F*^2^)] = 0.052	H-atom parameters constrained
*wR*(*F*^2^) = 0.118	*w* = 1/[σ^2^(*F*_o_^2^) + (0.0643*P*)^2^] where *P* = (*F*_o_^2^ + 2*F*_c_^2^)/3
*S* = 0.99	(Δ/σ)_max_ < 0.001
2439 reflections	Δρ_max_ = 0.56 e Å^−^^3^
182 parameters	Δρ_min_ = −0.58 e Å^−^^3^
Primary atom site location: structure-invariant direct methods	Extinction correction: none

### Special details

**Table:**

Geometry. All e.s.d.'s (except the e.s.d. in the dihedral angle between two l.s. planes) are estimated using the full covariance matrix. The cell e.s.d.'s are taken into account individually in the estimation of e.s.d.'s in distances, angles and torsion angles; correlations between e.s.d.'s in cell parameters are only used when they are defined by crystal symmetry. An approximate (isotropic) treatment of cell e.s.d.'s is used for estimating e.s.d.'s involving l.s. planes.
Refinement. Refinement of *F*^2^ against ALL reflections. The weighted *R*-factor *wR* and goodness of fit *S* are based on *F*^2^, conventional *R*-factors *R* are based on *F*, with *F* set to zero for negative *F*^2^. The threshold expression of *F*^2^ > σ(*F*^2^) is used only for calculating *R*-factors(gt) *etc*. and is not relevant to the choice of reflections for refinement. *R*-factors based on *F*^2^ are statistically about twice as large as those based on *F*, and *R*- factors based on ALL data will be even larger.

### Fractional atomic coordinates and isotropic or equivalent isotropic displacement parameters (Å^2^)

**Table:**

	*x*	*y*	*z*	*U*_iso_*/*U*_eq_	
C1	0.7059 (6)	0.6449 (6)	0.4901 (5)	0.0464 (10)	
C2	0.5527 (5)	0.6225 (5)	0.6085 (5)	0.0419 (9)	
C3	0.3922 (6)	0.7068 (5)	0.6049 (4)	0.0400 (9)	
H3	0.2908	0.6855	0.6806	0.048*	
C4	0.3786 (6)	0.8204 (5)	0.4932 (5)	0.0458 (10)	
C5	0.5336 (6)	0.8403 (6)	0.3738 (5)	0.0475 (11)	
H5	0.5278	0.9107	0.2938	0.057*	
C6	0.6944 (6)	0.7556 (5)	0.3752 (5)	0.0413 (9)	
C7	0.5468 (6)	0.5007 (6)	0.7344 (5)	0.0476 (10)	
H7	0.4369	0.4830	0.7991	0.057*	
C8	0.8486 (5)	0.7881 (5)	0.2457 (4)	0.0335 (8)	
H8	0.8285	0.8662	0.1746	0.040*	
C9	0.8484 (6)	0.2595 (5)	0.9110 (5)	0.0494 (11)	
H9A	0.8402	0.3379	0.9766	0.059*	
H9B	0.8668	0.1507	0.9574	0.059*	
C10	0.6753 (5)	0.2848 (5)	0.8787 (4)	0.0425 (10)	
H10A	0.6616	0.1845	0.8492	0.051*	
H10B	0.5766	0.3124	0.9655	0.051*	
C11	0.2057 (6)	0.9079 (6)	0.4876 (5)	0.0494 (11)	
H11A	0.1131	0.8652	0.5667	0.074*	
H11B	0.1953	0.8923	0.3978	0.074*	
H11C	0.1980	1.0224	0.4946	0.074*	
Cl1	0.82655 (13)	0.75907 (13)	0.86577 (11)	0.0428 (3)	
Mn1	0.91488 (7)	0.43436 (7)	0.64841 (6)	0.0349 (2)	
N1	0.6784 (5)	0.4187 (4)	0.7614 (4)	0.0475 (9)	
N2	0.9957 (5)	0.2803 (4)	0.7770 (4)	0.0469 (9)	
O1	0.8573 (4)	0.5649 (4)	0.4947 (3)	0.0449 (7)	
O2	0.9409 (4)	0.6619 (4)	0.7542 (3)	0.0523 (8)	
O3	0.9142 (4)	0.8814 (4)	0.8771 (3)	0.0457 (7)	
O4	0.7776 (4)	0.6558 (4)	0.9924 (3)	0.0476 (7)	
O5	0.6808 (4)	0.8267 (4)	0.8254 (3)	0.0551 (9)	

### Atomic displacement parameters (Å^2^)

**Table:**

	*U*^11^	*U*^22^	*U*^33^	*U*^12^	*U*^13^	*U*^23^
C1	0.036 (2)	0.059 (3)	0.043 (2)	0.0015 (19)	−0.0149 (18)	−0.010 (2)
C2	0.037 (2)	0.039 (2)	0.049 (2)	−0.0074 (17)	−0.0145 (19)	−0.0029 (18)
C3	0.038 (2)	0.044 (2)	0.042 (2)	−0.0069 (17)	−0.0163 (17)	−0.0121 (17)
C4	0.052 (3)	0.041 (2)	0.050 (2)	0.0047 (19)	−0.028 (2)	−0.0122 (19)
C5	0.038 (2)	0.067 (3)	0.041 (2)	0.005 (2)	−0.0200 (19)	−0.014 (2)
C6	0.043 (2)	0.0331 (19)	0.045 (2)	−0.0020 (17)	−0.0115 (18)	−0.0112 (17)
C7	0.039 (2)	0.055 (3)	0.045 (2)	−0.012 (2)	−0.0046 (19)	−0.010 (2)
C8	0.042 (2)	0.0357 (19)	0.0332 (19)	−0.0088 (16)	−0.0277 (17)	0.0084 (15)
C9	0.036 (2)	0.042 (2)	0.058 (3)	0.0016 (18)	−0.009 (2)	0.008 (2)
C10	0.043 (2)	0.049 (3)	0.039 (2)	−0.0161 (19)	−0.0178 (19)	0.0065 (18)
C11	0.048 (3)	0.055 (3)	0.044 (2)	0.002 (2)	−0.016 (2)	−0.010 (2)
Cl1	0.0484 (6)	0.0439 (5)	0.0378 (5)	−0.0173 (4)	−0.0121 (4)	−0.0020 (4)
Mn1	0.0334 (3)	0.0348 (3)	0.0293 (3)	−0.0010 (2)	−0.0074 (2)	0.0043 (2)
N1	0.042 (2)	0.044 (2)	0.051 (2)	−0.0087 (17)	−0.0114 (17)	0.0014 (16)
N2	0.049 (2)	0.045 (2)	0.0361 (18)	0.0054 (17)	−0.0119 (16)	0.0038 (16)
O1	0.0312 (14)	0.0499 (17)	0.0430 (16)	0.0020 (12)	−0.0097 (12)	0.0100 (13)
O2	0.0483 (18)	0.0482 (17)	0.0523 (18)	−0.0217 (14)	0.0020 (15)	−0.0113 (14)
O3	0.0583 (18)	0.0484 (17)	0.0359 (15)	−0.0247 (14)	−0.0123 (13)	−0.0094 (12)
O4	0.0412 (16)	0.0527 (18)	0.0475 (17)	−0.0193 (13)	−0.0142 (13)	0.0133 (14)
O5	0.0497 (18)	0.0483 (18)	0.0445 (17)	0.0108 (14)	−0.0063 (14)	0.0157 (14)

### Geometric parameters (Å, °)

**Table:**

C1—O1	1.321 (5)	C9—H9B	0.9700
C1—C6	1.383 (6)	C10—N1	1.492 (5)
C1—C2	1.406 (6)	C10—H10A	0.9700
C2—C3	1.393 (6)	C10—H10B	0.9700
C2—C7	1.488 (6)	C11—H11A	0.9600
C3—C4	1.376 (6)	C11—H11B	0.9600
C3—H3	0.9300	C11—H11C	0.9600
C4—C5	1.417 (6)	Cl1—O4	1.394 (3)
C4—C11	1.499 (6)	Cl1—O5	1.405 (3)
C5—C6	1.390 (6)	Cl1—O3	1.406 (3)
C5—H5	0.9300	Cl1—O2	1.413 (3)
C6—C8	1.485 (6)	Mn1—N2	1.888 (3)
C7—N1	1.269 (6)	Mn1—N1	1.893 (4)
C7—H7	0.9300	Mn1—O1	1.900 (3)
C8—N2^i^	1.262 (5)	Mn1—O1^i^	1.909 (3)
C8—H8	0.9300	Mn1—O2	2.391 (3)
C9—N2	1.460 (5)	Mn1—Mn1^i^	2.9228 (11)
C9—C10	1.556 (6)	N2—C8^i^	1.262 (5)
C9—H9A	0.9700	O1—Mn1^i^	1.909 (3)
			
O1—C1—C6	121.9 (4)	C4—C11—H11B	109.5
O1—C1—C2	119.0 (4)	H11A—C11—H11B	109.5
C6—C1—C2	119.0 (4)	C4—C11—H11C	109.5
C3—C2—C1	119.4 (4)	H11A—C11—H11C	109.5
C3—C2—C7	116.3 (4)	H11B—C11—H11C	109.5
C1—C2—C7	124.0 (4)	O4—Cl1—O5	110.61 (18)
C4—C3—C2	122.3 (4)	O4—Cl1—O3	112.37 (19)
C4—C3—H3	118.8	O5—Cl1—O3	111.3 (2)
C2—C3—H3	118.8	O4—Cl1—O2	107.3 (2)
C3—C4—C5	117.5 (4)	O5—Cl1—O2	106.4 (2)
C3—C4—C11	122.3 (4)	O3—Cl1—O2	108.58 (18)
C5—C4—C11	119.9 (4)	N2—Mn1—N1	91.90 (16)
C6—C5—C4	120.6 (4)	N2—Mn1—O1	170.28 (14)
C6—C5—H5	119.7	N1—Mn1—O1	93.45 (14)
C4—C5—H5	119.7	N2—Mn1—O1^i^	93.60 (13)
C1—C6—C5	120.9 (4)	N1—Mn1—O1^i^	168.66 (16)
C1—C6—C8	123.0 (4)	O1—Mn1—O1^i^	79.78 (13)
C5—C6—C8	116.1 (4)	N2—Mn1—O2	93.10 (15)
N1—C7—C2	125.8 (4)	N1—Mn1—O2	97.47 (14)
N1—C7—H7	117.1	O1—Mn1—O2	94.23 (13)
C2—C7—H7	117.1	O1^i^—Mn1—O2	92.12 (12)
N2^i^—C8—C6	126.1 (3)	N2—Mn1—Mn1^i^	132.98 (11)
N2^i^—C8—H8	117.0	N1—Mn1—Mn1^i^	132.81 (12)
C6—C8—H8	117.0	O1—Mn1—Mn1^i^	40.01 (8)
N2—C9—C10	110.2 (4)	O1^i^—Mn1—Mn1^i^	39.77 (8)
N2—C9—H9A	109.6	O2—Mn1—Mn1^i^	94.14 (8)
C10—C9—H9A	109.6	C7—N1—C10	126.4 (4)
N2—C9—H9B	109.6	C7—N1—Mn1	125.2 (3)
C10—C9—H9B	109.6	C10—N1—Mn1	108.2 (3)
H9A—C9—H9B	108.1	C8^i^—N2—C9	125.4 (3)
N1—C10—C9	109.9 (3)	C8^i^—N2—Mn1	126.2 (3)
N1—C10—H10A	109.7	C9—N2—Mn1	108.3 (3)
C9—C10—H10A	109.7	C1—O1—Mn1	130.6 (3)
N1—C10—H10B	109.7	C1—O1—Mn1^i^	129.0 (3)
C9—C10—H10B	109.7	Mn1—O1—Mn1^i^	100.22 (13)
H10A—C10—H10B	108.2	Cl1—O2—Mn1	134.41 (17)
C4—C11—H11A	109.5		

Symmetry codes: (i) −*x*+2, −*y*+1, −*z*+1.
